# Central-line associated bloodstream infection (CLABSI) in patients hospitalized with COVID-19

**DOI:** 10.1017/ash.2022.116

**Published:** 2022-05-16

**Authors:** Minji Kang, Zane Conrad, Elizabeth Thomas, Doramarie Arocha, Julie Trivedi

## Abstract

**Background:** Significant increases in healthcare-associated infections (HAIs) including central-line associated blood stream infections (CLABSIs) have been reported during the COVID-19 pandemic. Acute-care hospitals have faced staffing and personal protective equipment shortages, increased critical care capacity, and diversion of resources from traditional HAI surveillance and prevention efforts. In this study, we characterized CLABSIs among patients with COVID-19 and compared demographics, comorbidities, and outcomes between patients diagnosed with CLABSI with and without COVID-19. **Methods:** This is an observational retrospective cohort study of all patients diagnosed with CLABSI as defined by NHSN at William P. Clements Jr. University Hospital from April 1, 2020, through September 30, 2021. A retrospective chart review was conducted to identify demographics, comorbidities, and outcomes of hospitalized patients diagnosed with CLABSI. Patients hospitalized with and without COVID-19 were compared using the independent-sample *t* test for means and the χ^2^ test for proportions. **Results:** Overall, 82 patients diagnosed with CLABSI between April 1, 2020, to September 30, 2021, among whom 31 (38%) were hospitalized with COVID-19 and 51 (62%) were not hospitalized with COVID-19. Patients hospitalized with COVID-19 were significantly more likely to be obese (58% for COVID-19 positive vs 26% for COVID-19 negative; *P* = .01) and to require extracorporeal membrane oxygenation (19% vs 4%; *P* = .04). However, COVID-19 patients were significantly less likely to have hematologic malignancy (7% vs 28%; *P* = .03), undergone bone marrow transplantation (0% vs 18%; *P* = .01), or have neutropenia (3% vs 22%; *P* = .03). There were no significant differences in line type or organism identified. Gram-positive pathogens were identified in 16 patients (52%) hospitalized with COVID-19. Gram-negative pathogens were identified in 3 patients (10%); fungal organisms were identified in 10 patients (32%), and 2 cases (7%) were polymicrobial. Patients with COVID-19 were significantly more likely to require an ICU stay (84% vs 43%). p **Conclusions:** High device utilization as well as prolonged hospitalization and line days among patients with COVID-19 along are contributing risk factors for CLABSI among patients hospitalized with COVID-19. This finding highlights the need for ongoing HAI surveillance and prevention efforts in patients hospitalized with COVID-19 given their characteristics and increased risk for CLABSI. Reinforcing infection prevention efforts by accentuating the importance of optimal line care and regular feedback are crucial, especially among patients hospitalized with COVID-19.

**Funding:** None

**Disclosures:** None

